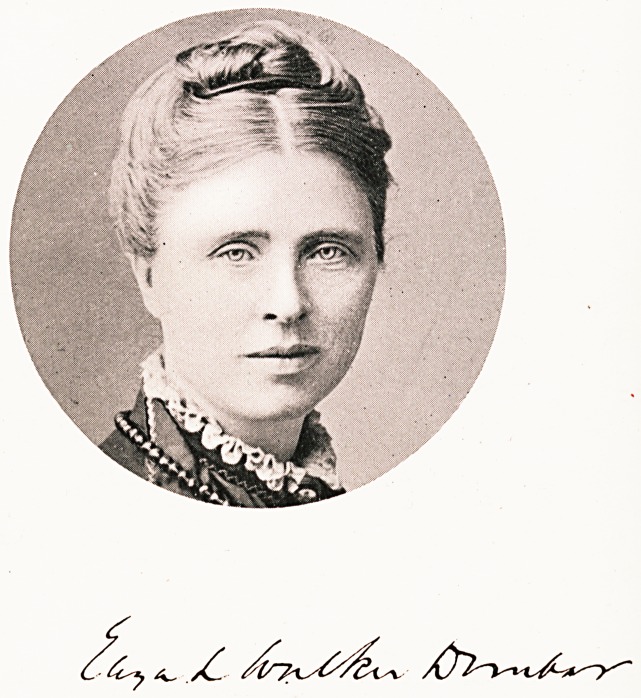# Eliza Walker Dunbar

**Published:** 1925

**Authors:** 


### ELIZA WALKER DUNBAR, M.D. Zürich, L.R.C.P.I., L.M.

Dr. Eliza Walker Dunbar, who we regret to hear died on August 25th in her eightieth year, was the pioneer among women medical practitioners in this city. A daughter of the late Alexander Walker, M.D., of the Bombay Military Department, she was born in India in 1845, and was educated at the Ladies' College, Cheltenham, and at Frankfort-on-Main. Having acquired a good knowledge of German, Miss Walker (who subsequently assumed the family name of Dunbar), decided, at the age of twenty-three, to study medicine at Zürich, and after four years spent at this University she obtained the degree of M.D. in 1872, on presentation of her thesis, " Embolie der Hirn-Arterien."

After a year's postgraduate work in Vienna Dr. Dunbar returned to England, acted for a time as House Surgeon to the Bristol Royal Hospital for Sick Children and Women, and then started practice in Dowry Square, Clifton, although debarred from British registration. When, after the passage of the Russell Gurney Enabling Act in 1876, the King and Queen's College of Physicians, Ireland, threw open its examinations to women, Dr. Dunbar, together with four other women, presented herself for examination, and obtained the licence of this College. She was thus enabled to enrol her name on the Medical Register of the United Kingdom.

Meanwhile, assisted by Miss Read and other supporters of the independence of women, Dr. Dunbar founded, in 1874, the Read Dispensary for Women and Children, Hotwells, and later, in 1895, with the co-operation of these and other friends, the Bristol Private Hospital for Women and Children was opened in Berkeley Square, Clifton. To both these institutions, founded with the double purpose of enabling women to practise medicine and surgery, and to facilitate the treatment of women by women, Dr. Dunbar gave many years of faithful and devoted service. She also acted for many years as Medical Officer to the Red Lodge Reformatory for Girls, and to the Bristol Training College for Elementary Teachers.

In 1906 she contributed to this *Journal* an article entitled, " The New Theory and Prophylactic Treatment of Puerperal Eclampsia."

Dr. Dunbar was essentially a fighter and a pioneer. She was intrepid in thought and action, and possessed, and retained to the end, high qualities of enterprise and courage. She was remarkably loyal and devoted to her old friends and patients, by whom also she was greatly beloved.

It is well to recall that the present advantage of freedom to work enjoyed by women are the direct fruit of the heroic struggles of those earlier determined spirits who, like Dr. Walker Dunbar, fought their way onward through obloquy and opposition. For many years Dr. Dunbar used to remove her brass plate at night lest morning should find it stolen or defaced !

Her death breaks another link with the past, but there remain the work done for the benefit of her successors and her fellow-citizens, and the influence for good exercised by a courageous spirit who fought well and aimed high.

## Figures and Tables

**Figure f1:**